# Reduction of Turbidity of Water Using Locally Available Natural Coagulants

**DOI:** 10.5402/2011/632189

**Published:** 2011-12-19

**Authors:** Md. Asrafuzzaman, A. N. M. Fakhruddin, Md. Alamgir Hossain

**Affiliations:** ^1^Department of Environmental Sciences, Jahangirnagar University, Savar, Dhaka 1342, Bangladesh; ^2^Microbiology and Chemical Division, Dhaka Water Supply and Sewerage Authority (DWASA), Asad Gate, Mohammadpur, Dhaka 1207, Bangladesh

## Abstract

Turbidity imparts a great problem in water treatment. *Moringa oleifera*, *Cicer arietinum*, and *Dolichos lablab* were used as locally available natural coagulants in this study to reduce turbidity of synthetic water. The tests were carried out, using artificial turbid water with conventional jar test apparatus. Optimum mixing intensity and duration were determined. After dosing water-soluble extracts of *Moringa oleifera, Cicer arietinum*, and *Dolichos lablab* reduced turbidity to 5.9, 3.9, and 11.1 nephelometric turbidity unit (NTU), respectively, from 100 NTU and 5, 3.3, and 9.5, NTU, respectively, after dosing and filtration. Natural coagulants worked better with high, turbid, water compare to medium, or low, turbid, water. Highest turbidity reduction efficiency (95.89%) was found with *Cicer arietinum*. About 89 to 96% total coliform reduction were also found with natural coagulant treatment of turbid water. Using locally available natural coagulants, suitable, easier, and environment friendly options for water treatment were observed.

## 1. Introduction

Water is undoubtedly the most vital element among the natural resources. In many developing countries, access to clean and safe water is a crucial issue. More than six million people die because of diarrhea which is caused by polluted water. Developing countries pay a high cost to import chemicals for water treatment [1]. This problem is critical in Bangladesh. About more than 80% of people in Bangladesh lack clean, safe water [2]. In the case of Dhaka, the capital city of over 10 million city dwellers, due to rapid urbanization and migration from rural areas there is a tremendous load on water consumption in the city [3]. The water condition of the surface water of Dhaka region has become highly polluted due to indiscriminate discharge of untreated waste from tannery, textile, and other industries, municipal waste into water bodies, poor drainage system, population increasing and urban encroachment, and river bank erosion, Hossain [4].

Water from all sources must have some form of purification before consumption. Various methods are used to make water safe and attractive to the consumer. The method employed depends on the character of the raw water. One of the problems with treatment of surface water is the large seasonal variation in turbidity, McConnachie et al. [5]. For the treatment of surface water, some traditional chemicals are used during the treatment of surface water at its various steps. Commonly used chemicals for various treatment units are synthetic organic and inorganic substances. In most of the cases, these are expensive since they are required in higher dose and do not show cost effectiveness. Many of the chemicals are also associated with human health and environmental problems, Kaggwa [6]. So, there raised a voice to develop cost-effective, easier, and environmental friendly process of water clarification.

The history of the use of natural coagulants is long. Natural organic polymers have been used for more than 2000 years in India, Africa, and China as effective coagulants and coagulant aids at high water turbidities. They may be manufactured from plant seeds, leaves, and roots [7]. These natural organic polymers are interesting because, comparative to the use of synthetic organic polymers containing acrylamide monomers, there is no human health danger and the cost of these natural coagulants would be less expensive than the conventional chemicals alike since it is locally available in most rural communities of Bangladesh. A number of effective coagulants from plant origin have been identified: *Nirmali*,* Okra*, red bean, sugar and red maize [8], *Moringa oleifera *[9], *Cactus latifera,* and seed powder of **Prosopis * juliflora *[10]. Natural coagulants have bright future and are concerned by many researchers because of their abundant source, low price, environment friendly, multifunction, and biodegradable nature in water purification.

The aims of the present study were

1. to reduce the level of turbidity and bacteriological contaminants from water using locally available natural coagulants,
2. to make the water treatment process easier and environmental friendly for household applications.

## 2. Materials and Methods

**Figure 1:**
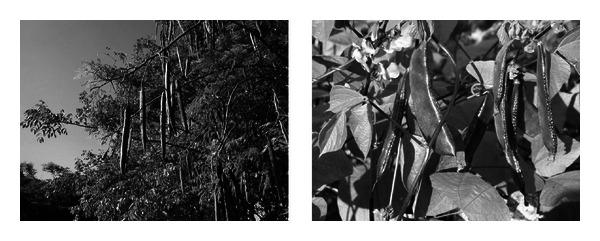
*Moringa oleifera *tree with pods and *Dolichos lablab* tree with pods.

Experiments were carried out in the laboratory of Dhaka Water Supply and Sewerage Authority (DWASA). *Moringa oleifera* (Sajina), *Dolichos lablab* (beans), and *Cicer arietinum* (dal) seeds used in this study were obtained from the Jahangirnagar University, Kanchanpur (Khulna) and local market of Dhaka city, respectively. The pictures of their seeds have been shown in Figure 1.

All coagulation experiments were carried out using synthetic artificial turbid water. A conventional jar test apparatus was used in the experiments to coagulate sample of synthetic turbid water using coagulants.

### 2.1. Preparation of Synthetic Water

Synthetic turbid water for the jar tests was prepared by adding clay materials to tap water. About 30 g of the clay materials was added to 1 liter of tap water. The suspension was stirred for about 1 hour to achieve a uniform dispersion of clay particles. Then it was allowed to settle for at least 24 hours for complete hydration of the clay materials. The supernatant suspension of synthetic turbid water was added to the sample water to achieve the desired turbidity just before coagulation.

### 2.2. Stock Solution of Natural Coagulants

*Moringa oleifera* seed pods are allowed to mature and dry naturally to a brown color on the tree. The seeds were removed from the pods, kept for sun dry, and external shells were removed. Mature seeds showing no signs of discoloration, softening, or extreme desiccation were used. The seed kernels were ground to fine powder using a kitchen blender to make it of approximate size of 600 *μ*m to achieve solubilization of active ingredients in the seed. Powder of *Cicer arietinum *(commercial name bashion) was bought from local market of Dhaka city. The grains of powder were maintained approximate size less than 600 *μ*m to achieve solubilization of active ingredients in the seed. Mature seeds of *Dolichos lablab* were used in the study. After sun dry, external shells were removed and seed kernel were obtained. Using grinder, fine powder achieved from seed kernel.

Distilled water was added to the powder to make 1% suspension of it. The suspension was vigorously shaken for 45 minutes using a magnetic stirrer to promote water extraction of the coagulant proteins, and this was then passed through filter paper (Whatman no. 42, 125 mm dia.). The filtrate portions were used for required dose of natural coagulants. Fresh solutions were prepared daily and kept refrigerated to prevent any ageing effects (such as change in pH, viscosity, and coagulation activity). Solutions were shaken vigorously before use.

### 2.3. Jar Test Operations

**Figure 2:**
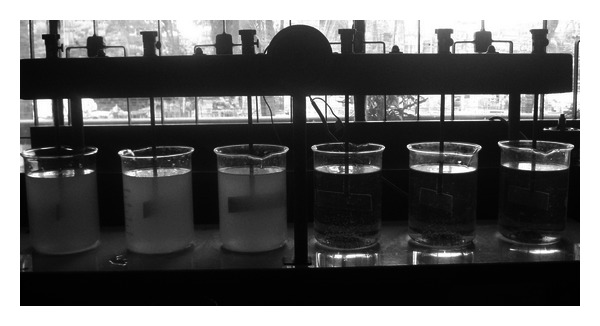
A conventional jar test apparatus for treatment of turbid water by natural coagulants.

Jar test is the most widely used experimental methods for coagulation-flocculation. A conventional jar test apparatus was used in the experiments to coagulate sample of synthetic turbid water using some coagulants (Figure 2). It was carried out as a batch test, accommodating a series of six beakers together with six-spindle steel paddles. Before operating the jar test, the sample was mixed homogenously. Then, the samples ought to be measured for turbidity, coliform count for representing an initial concentration. Coagulants of varying concentrations were added in the beakers. The whole procedures in the jar test were conducted in different rotating speed.

After the desired amount of coagulants was added to the suspensions, the beakers were agitated at various mixing time and speed, which consist of rapid mixing (200–250 rotation per minute, rpm) for 1–3 minutes and slow mixing (30–40 rpm) for 12–15 minutes. After the agitation being stopped, the suspensions were allowed to settle for 20–60 minutes. Finally, a sample was withdrawn using a pipette from the middle of supernatant for physicochemical and bacteriological measurements which represent the final concentration. All tests were performed at an ambient temperature in the range of 26–32°C and for different turbid ranges—higher (90–120) NTU, medium (40–50) NTU, and lower (25–35) NTU. In the experiment, the study was conducted by varying a few experimental parameters, which were coagulant dosage and mixing time in order to study their effect in flocculation and obtain the optimum condition for each parameter.

### 2.4. Analytical Methods

Turbidity is one of the important aesthetic properties of potable water, and it is also very useful in defining drinking water quality. Turbidity was measured using turbidity meter (Model-2100 P, HACH, USA). The pH of water was measured by using a pH meter (Model-sensION2, HACH, USA). The membrane filter (MF) technique can be used to test relatively large amount of samples and yield results more speedily than the multiple tube technique. Dehydrated m-ENDO (Difco, USA) media were used for the detection and quantification of total coliforms.

## 3. Results and Discussion

### 3.1. Reduction of Turbidity Using Natural Coagulants

**Figure 3:**
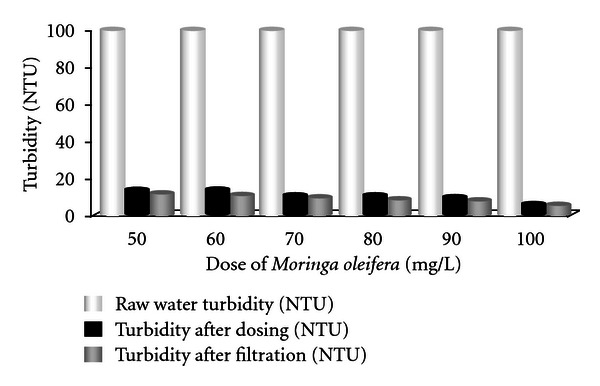
Removal of turbidity using various doses of *Moringa oleifera* (for highly turbid water).

**Figure 4:**
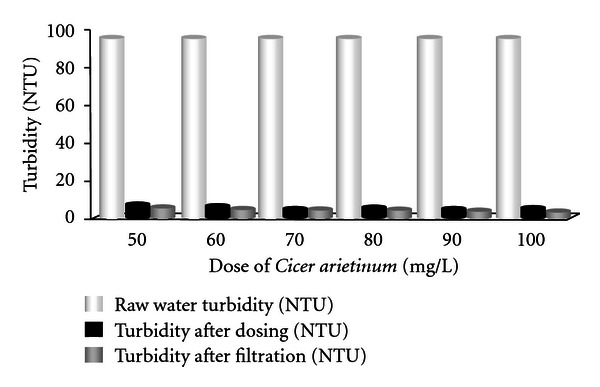
Removal of turbidity using various doses of **Cicer arietinum ** (for highly turbid water).

**Figure 5:**
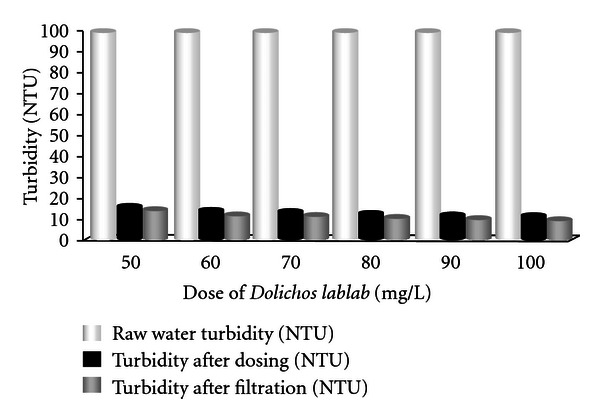
Removal of turbidity using various doses of* Dolichos lablab* (for highly turbid water).

The jar test operations using different coagulants were carried out in different turbidity ranges namely higher- (90–120) NTU, medium- (40–50) NTU, and lower- (25–35) NTU of synthetic turbid water. The efficiency of the extracts of *Moringa oleifera*,* Cicer arietinum*, and *Dolichos lablab* made them used as natural coagulants for the clarification of water. Doses started from 50 mg/L to 100 mg/L for corresponding six beakers. Turbidity was measured before and after treatment. Figures 3–5 show the results of different doses of coagulant treatment in jar test. From Figure 3, it is found that the raw water turbidity was 100 NTU. Turbidity reduced to 13.1, 12.7, 10.6, 10, 9.2, and 5.9 NTU corresponding to 50, 60, 70, 80, 90, and 100 mg/L *Moringa oleifera *doses respectively. After filtration, turbidity reduced to 11.2, 10.9, 9.1, 8.6, 7.9, and 5 NTU, respectively. For medium-turbidity water (turbidity 48 NTU), same doses reduce turbidity to 16.5, 16.1, 15.7, 15.1, 14.9, and 14.7 NTU, respectively, after dosing. And, after filtration, it was 14.1, 13.8, 13.5, 12.9, 12.8, and 12.6 NTU, respectively. *Moringa oleifera *work well in higher-turbidity water than lower- and medium-turbidity water. Turbidity reduction increases with increasing doses.

A similar study conducted by [11] showed that the processed *Moringa oleifera *was improved by isolation of bioactive constituents from the seeds as coagulant/flocculants which gave turbidity removal from 43.9, 91, and 333 NTU to 1.99, 1.40, and 2.20 NTU, respectively, corresponding to the of 0.05, 0.15, 0.30 mg/L. Kebreab et al. [12] found that the *Moringa oleifera* seed is nontoxic and good coagulant in water treatment. It is recommended to be used as a coagulant in developing countries. Encouraged by results of these studies, many developing countries have turned to use this plant as a viable coagulant in water and wastewater treatment on a small scale [13].

Results for the removal of turbidity using various doses of *Cicer arietinum *are shown in Figure 4. It was found that the raw water turbidity was 95 NTU. Turbidity reduced to 5.9, 5.1, 4.6, 4.5, 4.3, and 3.9 NTU corresponding to 50, 60, 70, 80, 90, and 100 mg/L *Cicer arietinum *doses. After filtration, turbidity reduced to 5, 4.3, 3.9, 3.8, 3.6, and 3.3 NTU, respectively. For medium-turbidity water (turbidity 49 NTU) same doses reduce turbidity to 12.6, 12.4, 10.2, 9.3, 9.1, and 9 NTU, respectively, after dosing. And, after filtration, it was 10.8, 10.6, 8.7, 7.9, 7.8, and 7.7 NTU, respectively. Most of the results using *Cicer arietinum *for higher-, medium-, and lower-turbidity-range comply with the Bangladesh drinking standard and the WHO guidelines [14, 15]. *Cicer arietinum* was found most effective for coagulation when the dose were 100 mg/L for high-, medium-, and low-turbidity water at a 3-min slow mixing time, 12 min slow mixing, and 30 min settling time. *Cicer arietinum *is cheap, easily cultivable, and available in Bangladesh. On the other hand naturally occurring coagulants are biodegradable and presumed safe for human health.

Results for the removal of turbidity using various doses of *Dolichos lablab *are shown in Figure 5. Different doses were used for different turbidity ranges, and turbidity was measured after dosing. From Figure 5, it is found that the raw water turbidity was 100 NTU. Turbidity reduced to 15.5, 14, 13.4, 12.3, 11.6, and 11.1 NTU corresponding to 50, 60, 70, 80, 90, and 100 mg/L *Dolichos lablab *doses. After filtration, turbidity reduced to 13.3, 12, 11.5, 10.5, 9.9, and 9.5 NTU, respectively. For medium-turbidity water (turbidity 49 NTU), same doses reduce turbidity to 17.1, 16.7, 16.3, 15.9, 15.8, and 15.6 NTU, respectively after, dosing. After filtration it was 14.7, 14.3, 14, 13.6, 13.5, and 13.4 NTU, respectively.

 A study was conducted using *Dolichos lablab *as natural coagulant for reduction of turbidity by Unnisa et al. [16], and the study showed that initial turbidities of 20 (low), 40 (medium), and 80 (high) NTUs mainly considerably decreased when the coagulant doses increased. Coagulation was the most effective at a dose of 200 mg/500 mL, when the coagulation activity of the *Dolichos lablab* seed extract was 65, 62, and 68% at a 60 min settling time. So the use of locally available materials like beans provides a better option for clean, safe water accessible to rural people.

Higher turbidity is a great problem of peripheral rivers of the Dhaka City rivers especially in rainy season, Hossain et al. [17]. Traditional alum and polyacryl amide (PAA) are used for the reduction of turbidity. Some problems are associated with the use of these chemicals. So, natural coagulants might bring a fruitful result in water treatment processes. Natural coagulants have been used to treat water for domestic household use for centuries in rural areas. Interest in the use of natural coagulants has increased over time, especially to reduce water and wastewater treatment problems in developing countries to avoid health risks [10].

### 3.2. Turbidity Reduction Efficiency of Different Coagulants in Different Turbidity Ranges

A comparative study of turbidity reduction efficiency of different coagulants in different turbidity ranges are presented in Table 1. In every case 50 to 100 mg/L doses were used. It was found that *Cicer arietinum* reduced maximum turbidity among all coagulants used. It reduced up to 95.89% for highly turbid water which is almost as same as the reduction capacity of alum. So, it was found most efficient among the studied natural coagulants. Second highest among the natural coagulants used for the study was for *Moringa oleifera.* It reduced up to 94.1% for highly turbid water. All of studied natural coagulants were efficient in higher-turbidity ranges than lower- and medium-turbidity waters.

The study also showed that higher dosages did not significantly increase pollutant removal and were not economically viable. Another study using Chitosan, a natural linear biopolyaminosaccharide, was obtained by alkaline deacetylation of chitin and showed turbidity reduction efficiency 74.3–98.2% at a pH 7.0–7.5, Mehdinejad et al. [18], and 94.9%, Hassan [19]. Previous study by [11] showed that the processed *Moringa oleifera *was improved by isolation of bioactive constituents from the seeds as a coagulant/flocculants which gave turbidity removal up to 99.3%. Another study regarding *Moringa oleifera* showed the effectiveness of *Moringa oleifera* for turbidity removals of up to 97% for high, turbid water and lower removals of 86% for low-turbidity waters, Abaliwano et al. [20]. So, these natural coagulants (*Cicer arietinum*,* Moringa oleifera*, and *Dolichos lablab*) might be considered as excellent alternative of traditional chemicals like alum and very efficient coagulants for high-turbidity ranges.

### 3.3. Reductions of Total Coliforms in Raw Water Using Natural Coagulants

Total coliform counts were determined for the turbid raw water and clarified treated water; the results are shown in Table 2. Very significant removal of total coliforms was found after treatment with natural coagulants. In the synthetic turbid water, total coliform count was recorded 1.35 × 10^3^ cfu/100 mL in an experiment, and, after treatment with water soluble extract of *Moringa oleifera, *it was 5.4 × 10^1^. *Cicer arietinum *and *Dolichos lablab* reduced turbidity from 1.05 × 10^3^ cfu/100 mL to 1.0 × 10^2^ cfu/100 mL and 1.1 × 10^2^ cfu/100 mL, respectively. In this experiment, the reduction of total coliform counts were about 96%, 90.47%, and 89.52% using *Moringa oleifera*,* Cicer arietinum*, and *Dolichos lablab*, respectively (Table 2). Previous study by Suarez et al. [21] demonstrated the ability of a recombinant *Moringa oleifera* protein to decrease the viability of Gram-negative or Gram-positive bacterial cells and to mediate the aggregation of negatively charged particles in suspension, such as bacterial cells, clay, or silicate microspheres. A study conducted by Michael [22] showed efficient reduction (80% to 99.5%) for high-turbidity pathogenic surface water and produces an aesthetically clear supernatant, concurrently accompanied by 90.00% to 99.99% (1 to 4 log) bacterial reduction. This may be an indication bactericidal activity of these natural coagulants.

## 4. Conclusion

Using some locally available natural coagulants, for example, *Moringa oleifera*, *Cicer arietinum*,* Dolichos lablab*, significant improvement in removing turbidity and total coliforms from synthetic raw water was found. Maximum turbidity reduction was found for highly turbid waters. After dosing, water-soluble extract of *Moringa oleifera*,* Cicer arietinum*, and *Dolichos lablab *reduced turbidity to 5.9, 3.9, and 11.1 NTU, respectively, from 100 NTU and 5, 3.3, and 9.5 NTU, respectively after dosing and filtration. It was also found that these natural coagulants reduced about 89–96% of total coliforms. Among the natural coagulants used in this study for turbidity reduction,* Cicer arietinum *was found most effective. It reduced up to 95.89% turbidity from the raw turbid water.

## Figures and Tables

**Table 1:** Reduction efficiency of turbidity using different coagulants in different turbidity ranges.

Coagulants	Dose used (mg/L)	% of turbidity reduction (High-*turbidity water)	% of turbidity reduction (Medium-*turbidity water)	% of turbidity reduction (Low-*turbidity water)
* Moringa oleifera*	50	86.9	65.62	56
	60	87.3	66.45	57.2
	70	89.4	67.29	58
	80	90	68.54	58.8
	90	90.8	68.95	59.2
	100	94.1	69.37	60

*Cicer arietinum*	50	93.78	74.28	62.58
	60	94.63	74.69	64.51
	70	95.15	79.18	66.12
	80	95.26	81.02	70
	90	95.47	81.42	70.96
	100	95.89	81.63	71.29

*Dolichos lablab*	50	84.5	65.10	49.71
	60	86	65.91	51.42
	70	86.6	66.73	56.28
	80	87.7	67.55	57.14
	90	88.4	67.75	59.42
	100	88.9	68.16	60.85

*For *Moringa oleifera *(high turbidity = 100 NTU, medium turbidity = 48 NTU, low turbidity = 25 NTU); *Cicer arietinum *(high turbidity = 95 NTU, medium turbidity = 49 NTU, low turbidity = 31 NTU); *Dolichos lablab *(high turbidity = 100 NTU, medium turbidity = 49 NTU, low turbidity = 35 NTU), and for alum high turbidity was 100 NTU.

**Table 2:** Reduction of total coliform after treatment using natural coagulants.

Coagulants	Total coliform counts (cfu/100 mL)		% reduction
	Before treatment	After treatment	
*Moringa oleifera*	1.35 × 10^3^	5.4 × 10^1^	96
*Cicer arietinum*	1.05 × 10^3^	1.0 × 10^2^	90.47
*Dolichos lablab*	1.05 × 10^3^	1.1 × 10^2^	89.52

## References

[1] Ghebremichael KA (2004). *Moringa seed and pumice as natural alternative materials for drinking water treatment*.

[2] Gomes DJ Waterborne illness: a real disaster in Bangladesh.

[3] Khan AR (June 2000). Increasing environmental pollution in Dhaka city and plans for control. *Souvenir on the Occasion of the World Environment Day*.

[4] Hossain MA (2009). *Impact of ammonia contamination on surface water treatment processes and its removal from polluted river water by aquatic macrophytes and nitrifying bacteria*.

[5] McConnachie GL, Folkard GK, Mtawali MA, Sutherland JP (1999). Field trials of appropriate hydraulic flocculation processes. *Water Research*.

[6] Kaggwa RC, Mulalelo CI, Denny P, Okurut TO (2001). The impact of alum discharges on a natural tropical wetland in Uganda. *Water Research*.

[7] Kawamura S (1991). Effectiveness of natural polyelectrolytes in water treatment. *Journal of the American Water Works Association*.

[8] Gunaratna KR, Garcia B, Andersson S, Dalhammar G (2007). Screening and evaluation of natural coagulants for water treatment. *Water Science and Technology*.

[9] Jahn SAA (1988). Using Moringa seeds as coagulants in developing countries. *Journal of the American Water Works Association*.

[10] Diaz A, Rincon N, Escorihuela A, Fernandez N, Chacin E, Forster CF (1999). A preliminary evaluation of turbidity removal by natural coagulants indigenous to Venezuela. *Process Biochemistry*.

[11] Ali EA, Muyibi SA, Salleh HM, Salleh MRM, Alam MZ *Moringa oleifera* seeds as natural coagulant for water treatment.

[12] Ghebremichael AG, Gunaratna KR, Henriksson H, Brumer H, Dalhammar G (2005). A simple purification and activity assay of the coagulant protein from *Moringa oleifera* seed. *Water Research*.

[13] Ndabigengesere A, Narasiah KS, Talbot BG (1995). Active agents and mechanism of coagulation of turbid waters using *Moringa oleifera*. *Water Research*.

[14] Environmental Conservation Rules (ECR)

[15] WHO http://www.who.int/water_sanitation_health/dwq/gdwq0506.pdf.

[16] Unnisa SA, Deepthi P, Mukkanti K (2010). Efficiency studies with *Dolichos lablab* and solar disinfection for treating turbid waters. *Journal of Environmental Protection Science*.

[17] Hossain MA, Begum T, Fakhruddin ANM, Khan SI (2006). Bacteriological and physiochemical analyses of the raw3 and treated water of Saidabad water treatment plant, Dhaka. *Bangladesh Journal of Microbiology*.

[18] Mehdinejad MH, Bina B, Nikaeen M, Attar HM (2009). Effectiveness of chitosan as natural coagulant aid in removal of turbidity and bacteria from turbid waters. *Journal of Food, Agriculture and Environment*.

[19] Hassan MAA, Li TP, Noor ZZ (2009). Coagulation and flocculation treatment of wastewater in textile industry using chitosan. *Journal of Chemical and Natural Resources Engineering*.

[20] Aabliwano JK, Ghebremichael KA, Amy GL (2008). *Application of Purified Moringa oleifera Coagulant for Surface Water Treatment. UNESCO-IHE*.

[21] Suarez M, Entenza JM, Doerries C (2003). Expression of a plant-derived peptide harboring water-cleaning and antimicrobial activities. *Biotechnology and Bioengineering*.

[22] Michael L (2010). *Bioremediation of turbid surface water using seed extract from Moringa oleifera Lam. (Drumstick) tree*.

